# Supplementary material for: Development of mesothelioma-specific oncolytic immunotherapy enabled by immunopeptidomics of murine and human mesothelioma tumors
Source: Nat Commun. 2023 Nov 3;14:7056. doi: 10.1038/s41467-023-42668-7 (PMC10624665; doi:10.1038/s41467-023-42668-7)
Supplement: Supplementary file 1 — Supplementary Information [file 41467_2023_42668_MOESM1_ESM.pdf]

Supplementary Information

Title: Development of mesothelioma-specific oncolytic immunotherapy enabled by immunopeptidomics of murine and human mesothelioma tumors

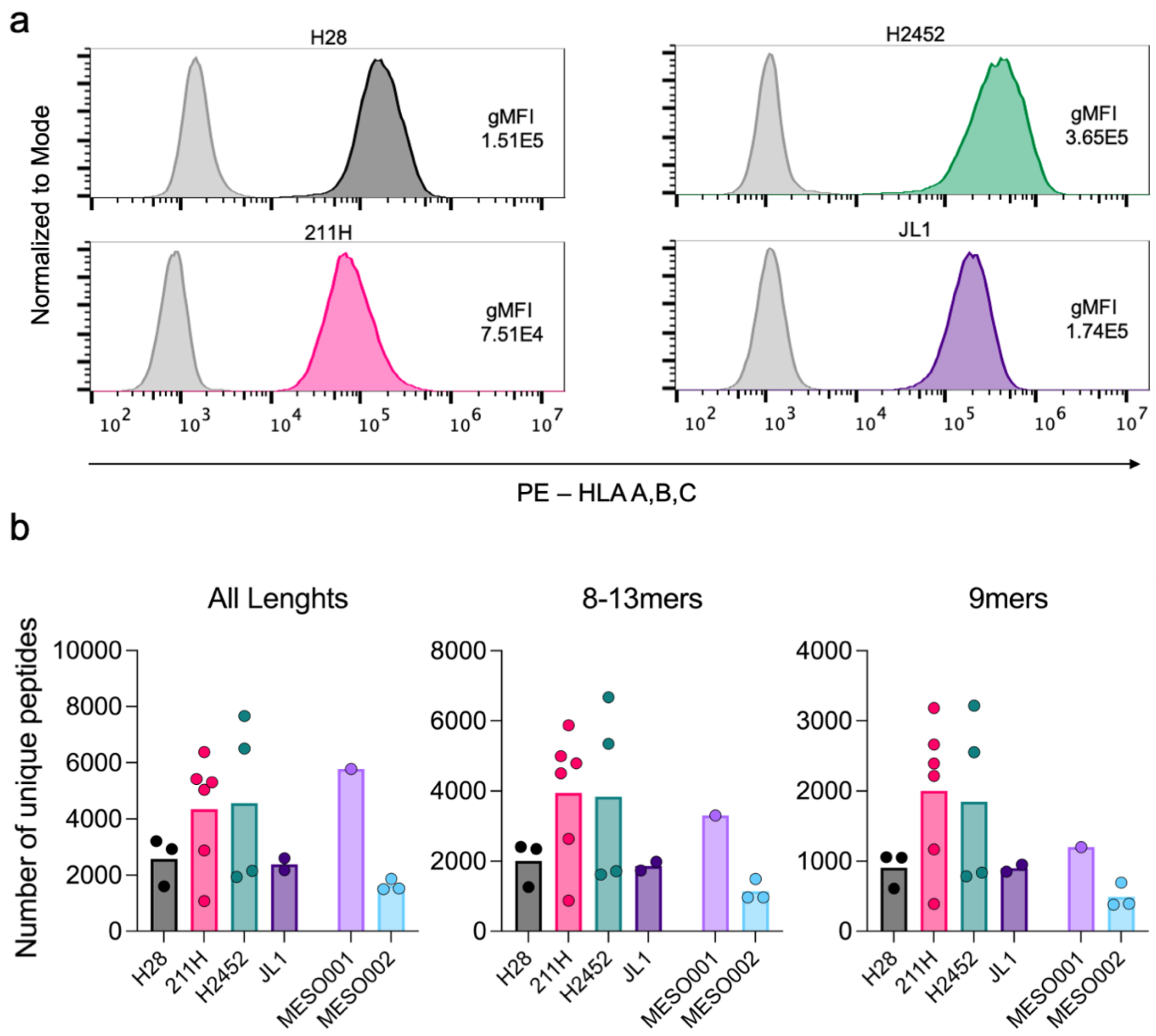

***Supplementary Fig 1**. A) Flow cytometry analysis of the human mesothelioma cell lines utilized in this study demonstrated high expression levels of MHC class I molecules on the cell surface. B) The number of unique eluted peptides from both human mesothelioma cell lines and patient-derived tumor samples. The displayed numbers represent the mean number of peptides across all lengths, as well as specifically for 8-13mers and 9mers. For H28 cells n=3, for 211H n=6, for H2452 n=4, for JL1 n=2, for MESO001 n=1, for MESO002 n=3.*

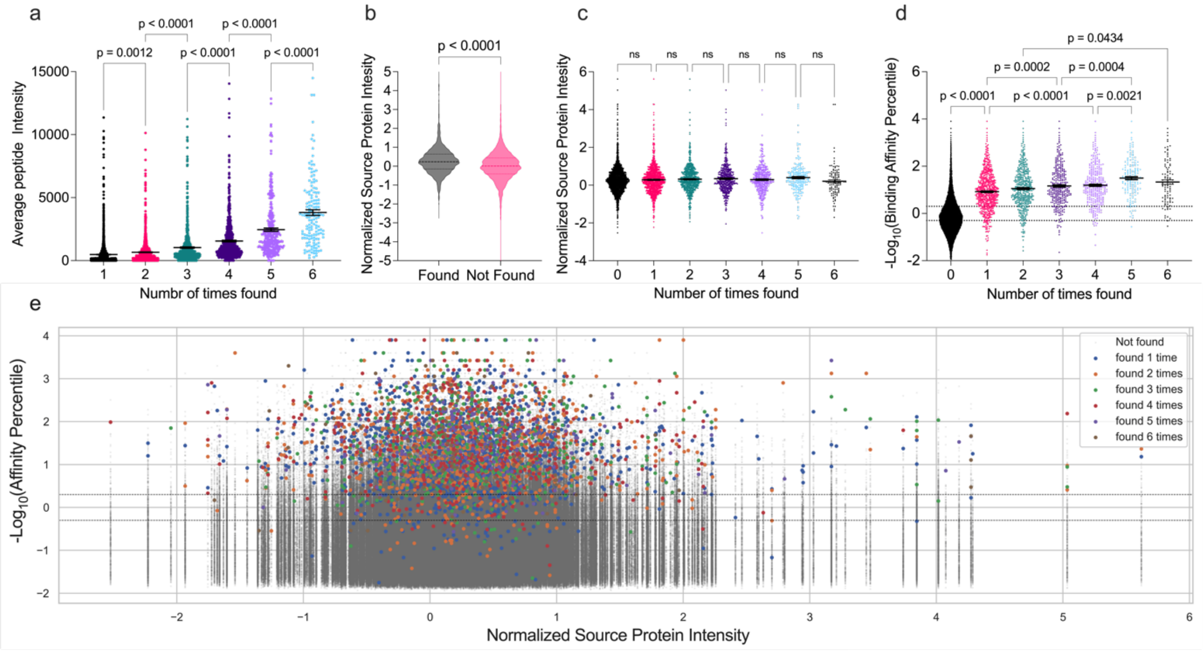

***Supplementary Figure 2**: Detailed analysis of the relationship between peptide:MHC binding affinity and source protein abundance in influencing the repertoire of MHC-presented peptides in MSTO-211H. A) Investigation of variations in average spectral intensities of peptides eluted in different replicates. For each independent replicate, mean ± SEM is shown. B) Comparison of protein levels between proteins with peptides "found" or "not found" in our MSTO-211H eluted peptide dataset. C) Examination of the correlation between protein abundance and the frequency of a specific peptide from that protein being found in our eluted peptide dataset. For each independent replicate, mean ± SEM is shown. D) Analysis of the relationship between peptide:MHC binding affinity and the frequency of occurrence for a particular peptide in our eluted peptide dataset. For each replicate, mean ± SEM is shown. E) Scatter plot demonstrating the association between predicted binding affinity for each peptide and the abundance of its corresponding source protein. Peptides not detected in our immunopeptidomics datasets are represented by grey dots, while colored dots represent peptides found 1 to 6 times. For all the data shown, statistical significance was determined using ordinary one-way ANOVA with Tukey correction. Source data are provided as a Source Data file.*

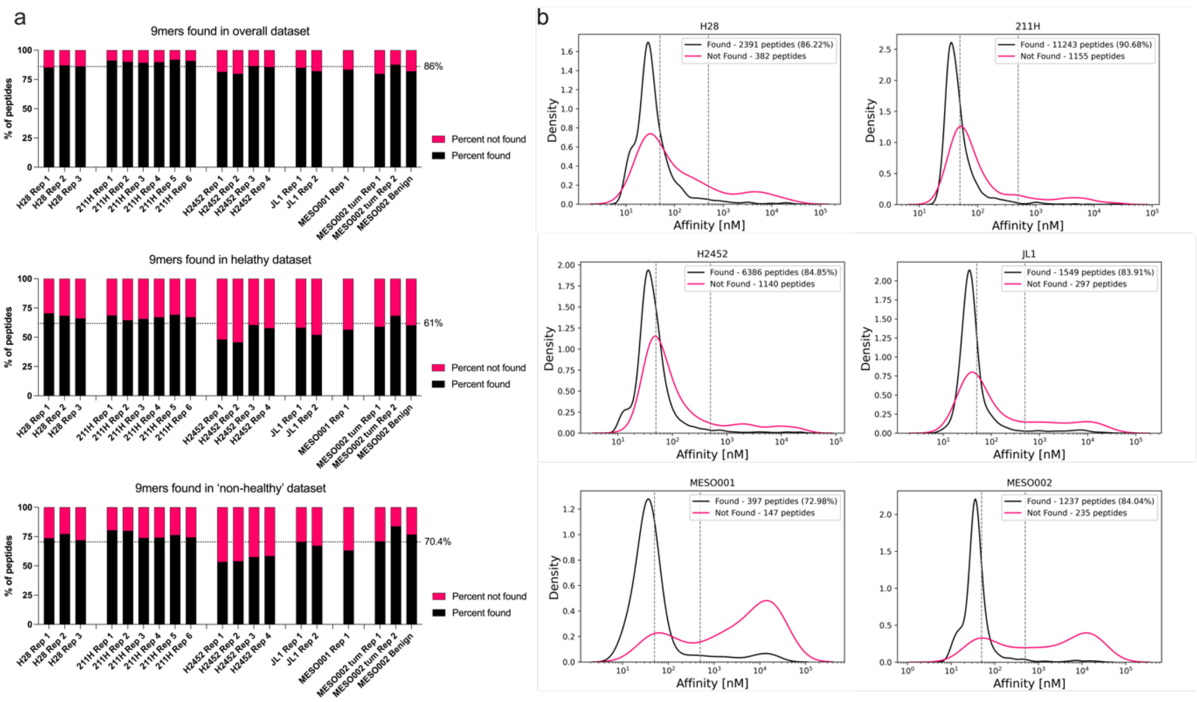

Supplementary Figure 3: *Analysis of previously characterized epitopes and peptide presentation profiles among the eluted peptides identified through immunopeptidomics. A) Percentage of peptides that have been previously characterized and are stored in the IEDB (Immune Epitope Database) repository. The results are presented for three different subsets: the entire database of characterized MHC ligands, a subset containing human peptides eluted from "healthy" samples, and a subset containing human peptides eluted from "non-healthy" samples. B) Comparison of the predicted MHC binding affinity profiles between peptides present in our dataset which were also found or not in the IEDB repository. The vertical grey dotted lines represent, from left to right, the affinity thresholds for "strong binders" and "weak binders," respectively. The number of independent samples for the data shown above was:* H28 n=3, 211H n=6, H2452 n=4, JL1 n=2, MESO001 n=1, MESO002 n=3

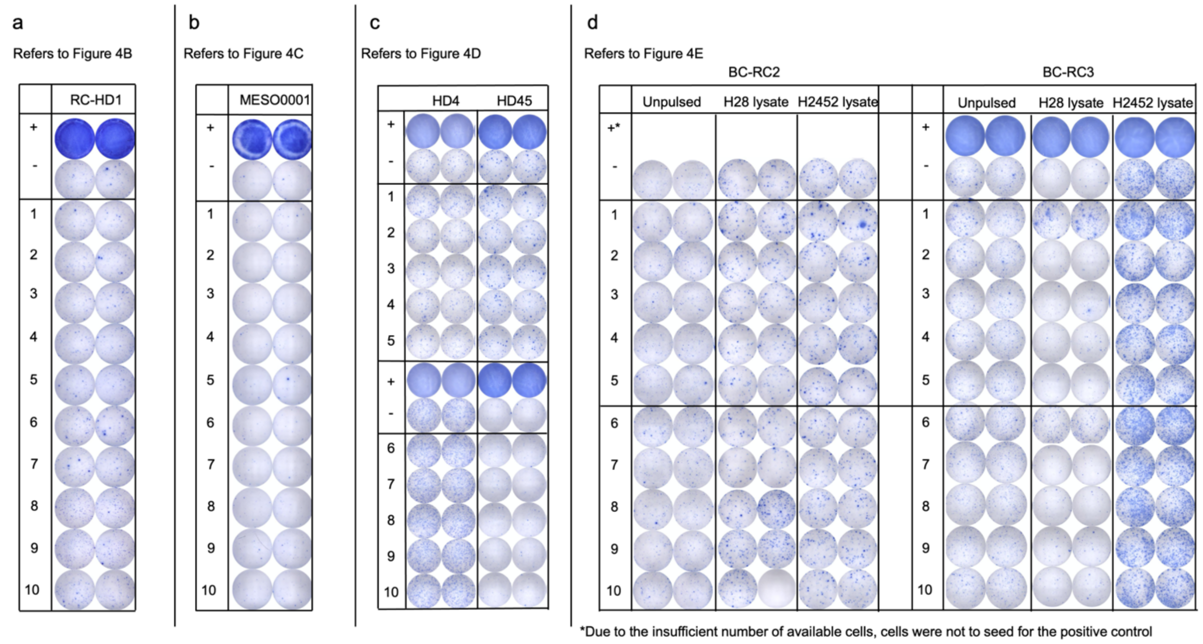

Supplementary Figure 4: Representative images of Human IFN-γ ELISpot data showed in Figure 4. *A) Representative wells pictures referring to the results presented in figure 4B. B) Representative pictures of ELISpot wells referring to the results presented in figure 4C. C) Representative pictures of ELISpot wells referring to the results presented in figure 4D. D) Representative pictures of ELISpot wells referring to the results presented in figure 4E.*

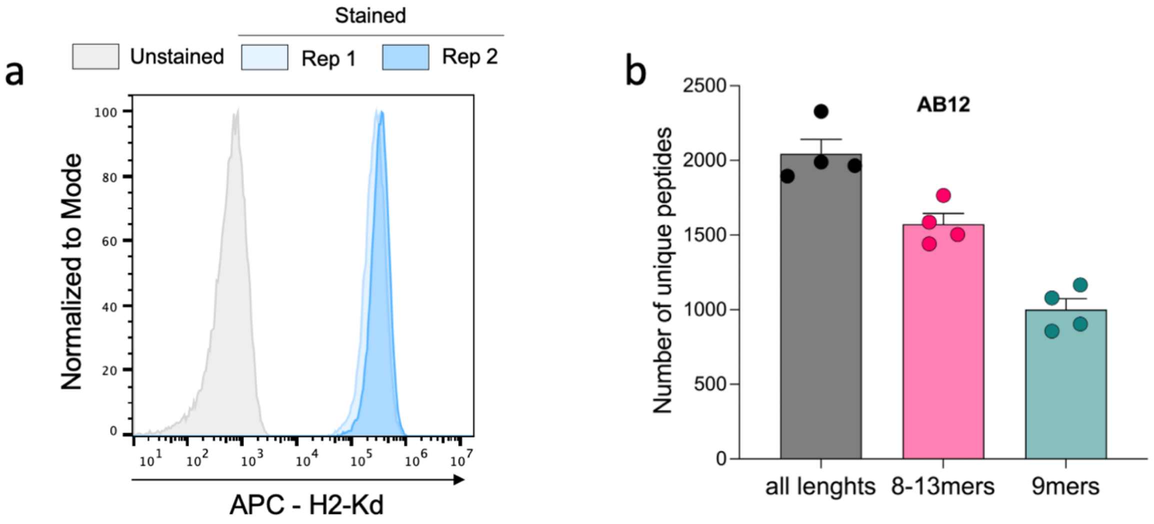

Supplementary Figure 5*: A) flow cytometry data showing MHC H2-Kd expression levels on the cell surface of AB12 murine mesothelioma cell line (unstained sample in grey, stained sample with anti-H2-Kd in blue). B) Bar plot showing mean number of eluted peptides* ±SEM *for the n=4 immunopeptidomics independent biological replicates for different peptide lengths intervals*

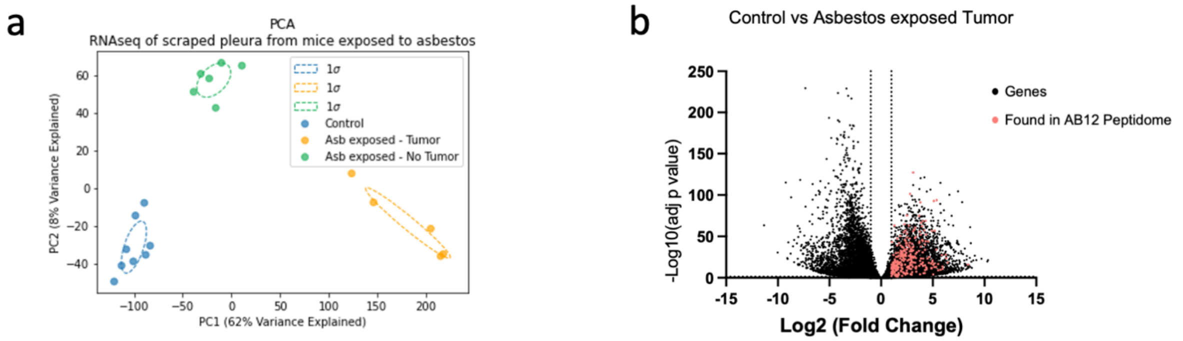

Supplementary Figure 6: *Reanalysis of the PRJEB15230 dataset to identify candidate epitopes for in vivo experimentation. A) Comparison of gene expression profiles among different samples: healthy mouse pleura (control, n=8), tumor samples from asbestos-exposed mice (Tumor, n=4), and pleura of mice exposed to asbestos but did not develop any tumors (No Tumor, n=6). The analysis focuses on identifying differences in gene expression between these groups. Ellipses around the datapoints represent one standard deviation of each distribution. B) Volcano plot illustrating the differential gene expression profile between the control group and tumor samples. Upregulated genes that correspond to peptides found in the AB12 immunopeptidomics analysis are highlighted in red.*

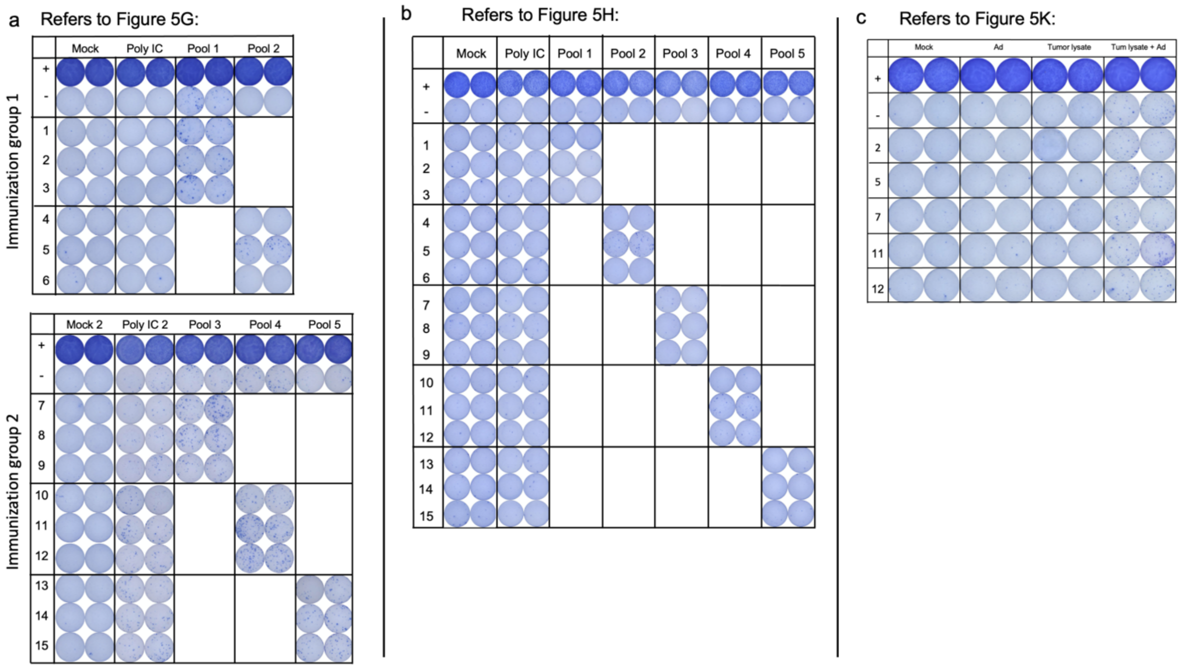

Supplementary Figure 7: Representative images of Murine IFN-γ ELISpot data showed in Figure 5. *A) Representative pictures of ELISpot wells referring to the results presented in figure 5G. B) Representative pictures of ELISpot wells referring to the results presented in figure 5H. C) Representative pictures of ELISpot wells referring to the results presented in figure 5K.*

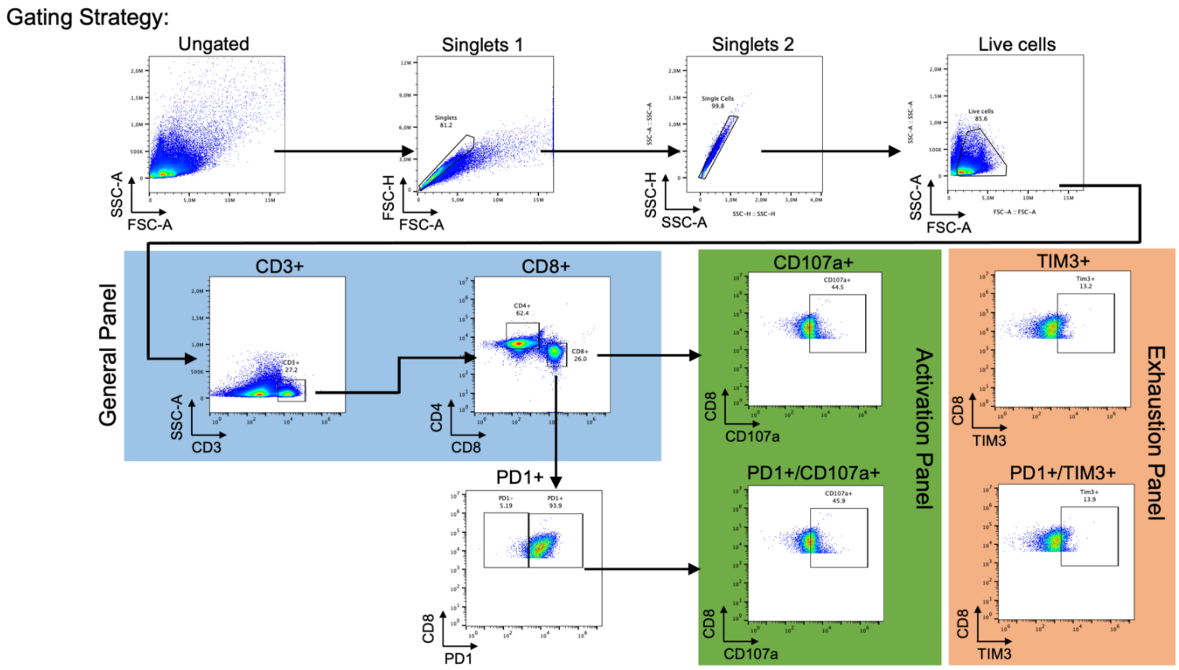

Supplementary Figure 8*: Schematic illustrating the gating strategy of flow cytometry data shown in Figure 6 and Supplementary Figure 9.*

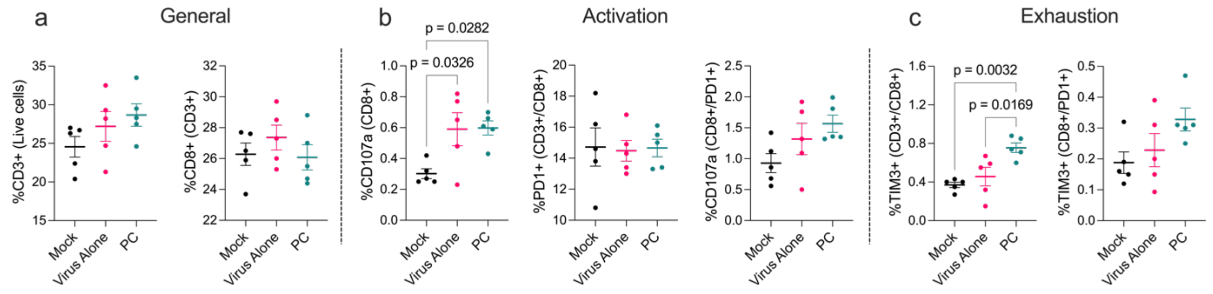

Supplementary Figure 9*: Immunological profile of secondary lymphoid tissues reveals T cell activation and exhaustion in mice treated with PeptiCRAd. Flow cytometry analysis was performed on spleenocytes (A-C). A) The frequency of CD3+ cells within the tumor and the frequency of CD8+ T cells. B) The frequency of CD8+ T cells positive for CD107a, PD1, and both CD107a and PD1. C) The frequency of CD8+ T cells positive for TIM3, and both TIM3 and PD1. All data are presented as dot plots showing the mean population frequency percentage* ±SEM *and each dot represent a single mouse in each treatment group (n=5). Statistical significance was determined using one-way ANOVA with Tukey's correction.*

***Supplementary table 1**: List of peptides identified through immunopeptidomics of tumor patients' samples and mesothelioma cell lines selected for immunogenicity screening.*

Peptide ID	Peptide Sequence	Source Gene Name	H28	H2452	211H	JL1	MESO001	MESO002	Times found overall	Allele specificity	Morani's dataset DE log2Fold Change	Found in Barone's dataset (1='Yes', 0='No')	Known T cell assay (IEDB) - Qualitative Measure
1	ALLNIKVKL	KRT18	0	2	0	0	1	0	3	HLA-A2	3,79	1	Negative, Positive
2	RLASYLDKV	KRT14, KRT19	2	2	0	1	1	1	7	HLA-A2	10,92	0	Negative, Positive
3	ALSDHHIYL	ALDOA	2	4	0	0	1	0	7	HLA-A2	1,46	1	Positive
4	YLLEKSRAI	MYH11	0	0	0	0	1	1	2	HLA-A2	5,15	0	Positive
5	TLFDYEVRL	UHRF1	2	2	0	0	1	1	6	HLA-A2	5,24	0	
6	ILLHLESEL	KRT18	0	0	2	0	1	0	3	HLA-A2	3,79	1	
7	ELRRTVQSL	KRT18	0	0	0	0	1	0	1	HLA-B8	3,79	1	
8	SIAKRLQSI	ALDOA	0	0	0	0	1	0	1	HLA-B8	1,46	1	
9	RLASYLDRV	KRT18	3	4	0	2	1	0	10	HLA-A2	3,79	1	
10	LRPSTSRSL	VIM	1	4	1	0	1	0	7	HLA-C6	3,97	0	

***Supplementary Table 2**: List of peptides identified through immunopeptidomics of the murine mesothelioma cell line AB12 selected for immunogenicity screening.*

Peptide ID	Gene Name	Accession	Peptide Sequence	Length	Rep 1	Rep 2	Rep 3	Rep 4	Found in Reps	Ensemble	baseMean
1	SF3B3	Q921M3-2	NYISGIQTI	9	1	1	1	1	4	ENSMUSG00000033732	875,57
2	MEN1	O88559-2	SYFKDRAHI	9	1	1	1	1	4	ENSMUSG00000024947	204,32
3	EDEM1	Q925U4	AYQSIQSYL	9	1	1	1	1	4	ENSMUSG00000030104	696,86
4	FRK	Q922K9	AYLESQNYI	9	1	1	1	1	4	ENSMUSG00000019779	87,09
5	SMC4	Q8CG47	FYFALRDTL	9	1	1	1	1	4	ENSMUSG00000034349	449,69
6	MTCH1	Q791T5-2	KYSGVLSSI	9	1	1	1	1	4	ENSMUSG00000024012	1522,36
7	YIPF5	Q9EQQ2	GYVYGISAI	9	1	1	1	1	4	ENSMUSG00000024487	356,92
8	E2F5	Q61502	SGPIHVLLI	9	1	1	1	1	4	ENSMUSG00000027552	65,26
9	WDR62	Q3U3T8-2	TYASTPSEI	9	1	1	1	1	4	ENSMUSG00000037020	55,06
10	FBXL6	Q9QXW0-2, Q9QXW0-3	TYSSQTTAI	9	1	1	1	1	2	ENSMUSG00000022559	159,4
11	DLG5	E9Q9R9	FYHTLHSRL	9	1	1	1	1	4	ENSMUSG00000021782	292,8
12	AP4M1	Q9JKC7	SFLPSGSEI	9	1	1	1	1	4	ENSMUSG00000019518	211,34
13	BUB1	O08901	SYGTLLNVI	9	1	1		1	3	ENSMUSG00000027379	39,54
14	EIF3C	Q8R1B4	TYSSVYDSI	9	1	1	1	1	4	ENSMUSG00000030738	3501,41
15	TOP2A	Q01320	TYIGSVELV	9	1	1	1	1	4	ENSMUSG00000020914	289,28

log2Fold Change	padj	-Log(adj p value)	MHCflurry Affinity percentile	MHCflurry Affinity	MHCflurry best allele	NetMHCpan Best Rank	NetMHCpan Allele Specificity	Same predicted allele? 1='Yes', 0='No'
1,98	1,38E-32	31,86	0,0011	29,62	H2-Kd	0,0020	H2-Kd	1
1,9	2,43E-29	28,61	0,0003	26,63	H2-Kd	0,0034	H2-Kd	1
3,11	4,70E-57	56,33	0,0003	26,68	H2-Kd	0,0023	H2-Kd	1
4,25	6,27E-16	15,20	0,0005	27,27	H2-Kd	0,0027	H2-Kd	1
3	6,02E-42	41,22	0,0058	33,71	H2-Kd	0,0055	H2-Kd	1
1,03	1,64E-16	15,78	0,0020	30,26	H2-Kd	0,0045	H2-Kd	1
1,43	2,25E-19	18,65	0,0189	42,36	H2-Kd	0,0099	H2-Kd	1
1,96	7,93E-16	15,10	0,0011	184,12	H2-Dd	0,0051	H2-Dd	1
2,04	3,65E-11	10,44	0,0020	30,16	H2-Kd	0,0044	H2-Kd	1
1,26	1,92E-12	11,72	0,0086	35,58	H2-Kd	0,0062	H2-Kd	1
1,93	1,58E-31	30,80	0,0100	36,28	H2-Kd	0,0075	H2-Kd	1
1,15	9,40E-19	18,03	0,0218	44,46	H2-Kd	0,0068	H2-Kd	1
4,87	9,11E-13	12,04	0,0136	38,48	H2-Kd	0,0084	H2-Kd	1
-0,76	0,00014	3,85	0,0049	32,94	H2-Kd	0,0089	H2-Kd	1
5,88	4,55E-28	27,34	0,0083	34,96	H2-Kd	0,0130	H2-Kd	1

Supplementary Table 3*. Peptides used through the animal study and in vitro experiments.*

#	Source Protein	Sequence	Quantity	Purity	Provider
	**Murine short peptides**				
1	SF3B3	NYISGIQTI	9mg	>85%	Chempeptide
2	MEN1	SYFKDRAHI	9mg	>85%	Chempeptide
3	EDEM1	AYQSIQSYL	9mg	>85%	Chempeptide
4	FRK	AYLESQNYI	9mg	>85%	Chempeptide
5	SMC4	FYFALRDTL	9mg	>85%	Chempeptide
6	MTCH1	KYSGVLSSI	9mg	>85%	Chempeptide
7	YIPF5	GYVYGISAI	9mg	>85%	Chempeptide
8	E2F5	SGPIHVLLI	9mg	>85%	Chempeptide
9	WDR62	TYASTPSEI	9mg	>85%	Chempeptide
10	FBXL6	TYSSQTTAI	9mg	>85%	Chempeptide
11	DLG5	FYHTLHSRL	9mg	>85%	Chempeptide
12	AP4M1	SFLPSGSEI	9mg	>85%	Chempeptide
13	FUT4	SYGTLLNVI	9mg	>85%	Chempeptide
14	GALE	TYSSVYDSI	9mg	>85%	Chempeptide
15	KDM5B	TYIGSVELV	9mg	>85%	Chempeptide
	**Murine polyK peptides**				
11	DLG5	KKKKKFYHTLHSRL	9mg	>95%	Genscript
12	AP4M1	KKKKKKKSFLPSGSEI	9mg	>95%	Genscript
	**Human peptides**				
1	KRT18	ALLNIKVKL	1-4mg	>90%	Genscript
2	KRT14, KRT19	RLASYLDKV	1-4mg	>90%	Genscript
3	ALDOA	ALSDHHIYL	1-4mg	>90%	Genscript
4	MYH11	YLLEKSRAI	1-4mg	>90%	Genscript
5	UHRF1	TLFDYEVRL	1-4mg	>90%	Genscript
6	KRT18	ILLHLESEL	1-4mg	>90%	Genscript
7	KRT18	ELRRTVQSL	1-4mg	>90%	Genscript
8	ALDOA	SIAKRLQSI	1-4mg	>90%	Genscript
9	KRT18	RLASYLDRV	1-4mg	>90%	Genscript
10	VIM	LRPSTSRSL	1-4mg	>90%	Genscript

Supplementary Table 4*: HLA typing of all the cell lines, healthy donors and patients' materials used in the current study.*

	HLA-A		HLA-B		HLA-C				
Samples	A1	A2	B1	B2	C1	C2	Source		
NCI-H28	68:01	02:02	35:118	53:02	04:01	06:02	ATCC		
NCI-H2452	01:01	02:01	57:01	35:01	04:01	06:02	ATCC		
MSTO-211H	01:01	03:01	07:02	39:01	07:02	12:03	ATCC		
JL1	03:01	02:05	49:01	55:01	03:04	07:01	ATCC		
MESO001	02:01	68:01	08:01	51:01	07:01	15:02	Helsinki University Hospital		
MESO002	01:01	02:01	07:02	08:01	07:01	07:02	Helsinki University Hospital		
	HLA-A		HLA-B		HLA-C				
#	A1	A2	B1	B2	C1	C2	Source	Disease status	Sample type
1	02:01	24:02	35:03	55:01	03:03	04:01	Humanitas Research Hospital	Healthy Donor	Buffy coat
2	02:01	01:01	08:01	40:01	03:01	03:01	Red Cross	Healthy Donor	Buffy coat
3	02:01	02:01	08:01	44:02	07:01	05:01	Red Cross	Healthy Donor	Buffy coat
4	02:01	02:01	08:01	56:01	01:02	07:01	Red Cross	Healthy Donor	Buffy coat
5	02:01	02:01	08:01	15:01	04:01	07:01	Red Cross Biobank	Healthy Donor	PBMCs
6	02:01	02:01	08:01	13:02	06:02	07:01	Red Cross Biobank	Healthy Donor	PBMCs
7	02:01	02:01	08:01	51:01	07:01	15:02	Red Cross Biobank	Healthy Donor	PBMCs
8	02:01	02:01	08:01	27:05	01:02	07:01	Red Cross Biobank	Healthy Donor	PBMCs
9	02:01	02:01	08:01	41:02	07:01	17:03	Red Cross Biobank	Healthy Donor	PBMCs
