# Supplementary material for: Development of mesothelioma-specific oncolytic immunotherapy enabled by immunopeptidomics of murine and human mesothelioma tumors
Source: Nat Commun. 2023 Nov 3;14:7056. doi: 10.1038/s41467-023-42668-7 (PMC10624665; doi:10.1038/s41467-023-42668-7)
Supplement: Supplementary file 3 — Reporting Summary [file 41467_2023_42668_MOESM3_ESM.pdf]

nature portfolio

Corresponding author(s): Vincenzo Cerullo

Last updated by author(s): Sep 12, 2023

Reporting Summary

Nature Portfolio wishes to improve the reproducibility of the work that we publish. This form provides structure for consistency and transparency in reporting. For further information on Nature Portfolio policies, see our Editorial Policies and the Editorial Policy Checklist.

Statistics

For all statistical analyses, confirm that the following items are present in the figure legend, table legend, main text, or Methods section.

n/a	Confirmed	
☐	☒	The exact sample size (*n*) for each experimental group/condition, given as a discrete number and unit of measurement
☐	☒	A statement on whether measurements were taken from distinct samples or whether the same sample was measured repeatedly
☐	☒	The statistical test(s) used AND whether they are one- or two-sided<br>*Only common tests should be described solely by name; describe more complex techniques in the Methods section.*
☒	☐	A description of all covariates tested
☐	☒	A description of any assumptions or corrections, such as tests of normality and adjustment for multiple comparisons
☐	☒	A full description of the statistical parameters including central tendency (e.g. means) or other basic estimates (e.g. regression coefficient) AND variation (e.g. standard deviation) or associated estimates of uncertainty (e.g. confidence intervals)
☐	☒	For null hypothesis testing, the test statistic (e.g. *F*, *t*, *r*) with confidence intervals, effect sizes, degrees of freedom and *P* value noted<br>*Give P values as exact values whenever suitable.*
☒	☐	For Bayesian analysis, information on the choice of priors and Markov chain Monte Carlo settings
☒	☐	For hierarchical and complex designs, identification of the appropriate level for tests and full reporting of outcomes
☒	☐	Estimates of effect sizes (e.g. Cohen's *d*, Pearson's *r*), indicating how they were calculated

Our web collection on statistics for biologists contains articles on many of the points above.

Software and code

Policy information about availability of computer code

Data collection	PEAKS Studio X+ (v10.5 build 20191016) was used for peptide sequence inference by spectral matching. Immunospot software installed on the Immunospot Series 6 Alpha ELISpot Analizer was used to acquire ELISpot data. BD Accuri C6 plus sampler software was used to acquire flow cytometry data.
Data analysis	Data analysis was performed using Graphpad Prism, Flowjo, Python. For some analysis of immunoeptidomics datasets a cusom script called PyptidomicsQC was produced and it is hosted on GitHub at the followng link https://github.com/JacopoChiaro/PyptidomicsQC.git

For manuscripts utilizing custom algorithms or software that are central to the research but not yet described in published literature, software must be made available to editors and reviewers. We strongly encourage code deposition in a community repository (e.g. GitHub). See the Nature Portfolio guidelines for submitting code & software for further information.

Data

Policy information about availability of data

All manuscripts must include a data availability statement. This statement should provide the following information, where applicable:

- Accession codes, unique identifiers, or web links for publicly available datasets
- A description of any restrictions on data availability
- For clinical datasets or third party data, please ensure that the statement adheres to our policy

All the mass spectrometry proteomics data have been deposited to the ProteomeXchange Consortium via the PRIDE partner repository under the identifier PXD038273. The publicly available murine mesothelioma gene expression data used in this study are available in the EBI repository database under accession code

PRJEB15230 [http://www.ebi.ac.uk/ena/data/view/PRJEB15230)]. The remaining data are available within the Article, Supplementary Information or Source Data file.

Research involving human participants, their data, or biological material

Policy information about studies with human participants or human data. See also policy information about sex, gender (identity/presentation), and sexual orientation and race, ethnicity and racism.

Reporting on sex and gender	Only two patients were involved in the current study: MESO001 and MESO002. Both the subjects are male, although the data or the results do not apply to only one gender. Sex and gender were not considered as inclusion or exclusion criterion during the study design. About the PBMCs or Buffy Coats derived by helathy donors obtained by the Finnish red cross Biobank, we didn't request to know the sex and the gender of the donors, hence this information is not reported in the manuscript.
Reporting on race, ethnicity, or other socially relevant groupings	As only two patients were involved in the current study (MESO001 and MESO002), both the Finns, and as the data or the results do not apply to specific ethnic groups, race and ethnicity were not considered as inclusion or exclusion criterion during the study design. Hence, they are not reported in the manuscript.
Population characteristics	Only two patients were involved in the current study (MESO001 and MESO002) bot the subjects are male and both are over 70 years of age. Both patients were diagnosed with low grade epithelioid mesothelioma. About the PBMCs or buffy coats derived by the healthy donors obtained by the Finnish Red Cross Biobank, we haven't requested any additional information but the HLA typing, for this reason, this latter is the only information reported in the article.
Recruitment	Patients were recruited under the Umbrella of iCAN cancer precision medicine flagship.
Ethics oversight	Patient's samples were received by the Helsinki University Hospital under the approval of the ethical review board (review number HUS/970/2021) and Helsinki University Hospital institutional review board (IRB) (approval HUS/8/2022).

Note that full information on the approval of the study protocol must also be provided in the manuscript.

Field-specific reporting

Please select the one below that is the best fit for your research. If you are not sure, read the appropriate sections before making your selection.

[x] Life sciences   [ ] Behavioural & social sciences   [ ] Ecological, evolutionary & environmental sciences

For a reference copy of the document with all sections, see nature.com/documents/nr-reporting-summary-flat.pdf

Life sciences study design

All studies must disclose on these points even when the disclosure is negative.

Sample size	No sample-size calculation was performed before any of the experiments. Sample size was selected for each experiment based on the on the previous knowledge gathered in years of experience and based on the general practices employed in our laboratory.
Data exclusions	The only excluded data belonged to the experimental part involving in vivo work. Exclusion criteria were the following: 1) Failed tumor engraftment. Whether a mouse never had developed the tumor after the tumor injection the mouse was excluded from the study. 2) Whether a mouse had to be euthanized for showing signs of discomfort, the mouse was excluded from the experiment.
Replication	We performed different number of biological replicates of the immunopeptidomics run for the human samples. We performed n=6 runs for the MSTO-211H, n=3 for the NCI-H28, n=4 for the NCI-H2452, n=2 for the JL1 based on the replication rate of the different cell lines and the ease of collecting enough material to perform the runs. For the MESO001 sample only 1 biological replicate was performed due to insufficient amount of biological material. Conversely, for the MESO002 sample, given the size of the tumor resection we were able to perform n=3 biological replicates. For peptide immunogenicity screening, n=5 healthy donors' PBMCs were tested only once via ELISpot assay. CD8+ T cells from n=4 Buffy Coats were expanded and restimulated using selected peptides described in the manuscript. T cell killing assay was performed only once for n=2 donors CD8 T cells due to insufficient amount of material.<br>For the murine cell line AB12, n=4 biological replicates for the immunopeptidomics assay were performed. In vivo peptide immunogenicity screening, Figure 5, was carried out with 3 independent experiments with at least three biological replicates for each group. The experiment assessing the capacity of the vaccination shown in figure 5 and 6 were performed only once given the large sample size (n=8 mice per group or more). Flow cytometry on murine tumor sample was performed only once using cells from five mice from each group (independent biological replicates). All the attempts at replication were successful.
Randomization	For in vitro experiments, in each experimental setting, all the samples were treated with all the conditions (controls). The order of the groups was randomized either in the placement in a multi well plate or in the acquisition. Samples for immunopeptidomics were collected across a long period of time. All the pellets required for running the assay were collected independently, at different time and in a random order. MHCs immunoaffinity purifications were performed on different days and using different columns which were randomly selected. Each column was used a maximum of 3 times per each specific sample). For in vivo experiments, when a lump was visible on the mouse flank after tumor cells injection, all mice of a cohort were transferred to a very large cage and were subsequently allocated in different cages until a number of maximum n=5 mice per cage was reached. In order to ensure that the average tumor size was comparable among different groups before any treatment and contrast the possibility that many big tumors would end up in a single group, mice with bigger tumors were allocated first followed by mice bearing progressively smaller tumors. Lastly, the group name and the corresponding therapy was randomly assigned to all the cages. Both 'group order' and 'mice order' were random at the moment of the treatment. The order of the treatment groups at the moment of the euthanasia was random. order for the sample collection and processing was also random.

Blinding | Blinding was not applied in this study as the experiments were conceptualized, executed and analyzed by the same person.

Reporting for specific materials, systems and methods

We require information from authors about some types of materials, experimental systems and methods used in many studies. Here, indicate whether each material, system or method listed is relevant to your study. If you are not sure if a list item applies to your research, read the appropriate section before selecting a response.

Materials & experimental systems

n/a	Involved in the study
☐	☒ Antibodies
☐	☒ Eukaryotic cell lines
☒	☐ Palaeontology and archaeology
☐	☒ Animals and other organisms
☒	☐ Clinical data
☒	☐ Dual use research of concern
☒	☐ Plants

Methods

n/a	Involved in the study
☒	☐ ChIP-seq
☐	☒ Flow cytometry
☒	☐ MRI-based neuroimaging

Antibodies

Antibodies used

Primary un-conjugated antobodies used for HLA immunoaffinity enrichment for immunopeptidomics assay:
inVivoMAb anti-mouse MHC Class I (H-2Kd, H-2Dd) (clone 34-1-2S, BioXCell, BE0180, Lebanon, NH, USA),
anti-human HLA-A, HLA-B, and HLA-C antibodies (inVivoMAb, clone W6/32, BioXCell, BE0180, Lebanon, NH, USA).

Primary conjugated antobodies used for flow cytometry assays:
APC anti-mouse CD3 (clone: 17A2, cat: 100236, Biolegend),
FITC anti-mouse CD8a (clone: 53-6.7, cat: 100706, Biolegend),
PerCP/cy5.5 anti-mouse CD107a (LAMP-1) (clone:1D4B, cat:121625, Biolegend),
PE anti-mouse CD279 (PD-1) (clone:29F.1A12, cat:135206, Biolegend),
PerCP/cy5.5 anti-mouse CD366 (Tim-3) (clone:RMT3-23, cat:119718, Biolegend),
PE-conjugated anti-human HLA-A, HLA-B, and HLA-C (clone W6/32, BioLegend 311406, San Diego, CA),
APC anti-mouse H2-Kd (clone:SF1-1.1, cat: 116619, Biolegend),
PE-Cy7 anti-CD107a (clone: H4A3, cat: 328617, Biolegend).

Validation

The validation for the inVivoMAb anti-mouse MHC Class I antibody can be found at the manufacturer website (https://bioxcell.com/invivomab-anti-mouse-mhc-class-i-h-2kd-h-2dd-be0180#tab_references) and in the following works:
Cleary, Simon J et al. “Complement activation on endothelium initiates antibody-mediated acute lung injury.” The Journal of clinical investigation vol. 130,11 (2020): 5909-5923. doi:10.1172/JCI138136;
Kapur R, Kim M, Shanmugabhavananthan S, Liu J, Li Y, Semple JW. C-reactive protein enhances murine antibody-mediated transfusion-related acute lung injury. Blood. 2015 Dec 17;126(25):2747-51. doi: 10.1182/blood-2015-09-672592. Epub 2015 Oct 9. PMID: 26453659.

The validation for the inVivoMAb anti-human HLA-A, HLA-B, and HLA-C antibodies can be found at the manufacturer website https://bioxcell.com/invivomab-anti-human-mhc-class-i-hla-a-hla-b-hla-c-be0079
Valenzuela, N M et al. “Blockade of p-selectin is sufficient to reduce MHC I antibody-elicited monocyte recruitment in vitro and in vivo.” American journal of transplantation : official journal of the American Society of Transplantation and the American Society of Transplant Surgeons vol. 13,2 (2013): 299-311. doi:10.1111/ajt.12016;
Valenzuela, Nicole M et al. “HLA class I antibodies trigger increased adherence of monocytes to endothelial cells by eliciting an increase in endothelial P-selectin and, depending on subclass, by engaging FcγRs.” Journal of immunology (Baltimore, Md. : 1950) vol. 190,12 (2013): 6635-50. doi:10.4049/jimmunol.1201434.

Validation for the APC anti-mouse CD3 antibody can be foundat the manufacturer website (https://www.biolegend.com/fr-lu/products/apc-anti-mouse-cd3-antibody-8055) and cited in the following works:
Radtke AJ, et al. 2020. Proc Natl Acad Sci U S A. 117:33455-65;
Radtke AJ, et al. 2022. Nat Protoc. 17:378-401.

Validation for the FITC anti-mouse CD8a antibody can be found at the manufacturer website (https://www.biolegend.com/fr-fr/products/fitc-anti-mouse-cd8a-antibody-153) and cited in the following works:
Shih FF, et al. 2006. J. Immunol. 176:3438;
Bouwer HGA, et al. 2006. P. Natl. Acad. Sci. USA 103:5102

Validation for the PerCP/cy5.5 anti-mouse CD107a (LAMP-1) antibody can be found at the manufacturer website (https://www.biolegend.com/nl-nl/products/purified-anti-mouse-cd107a-lamp-1-antibody-3585) and cited in the following works:
Roblek M, et al. 2022. Front Oncol. 12:777634;
Tersteegen A, et al. 2021. Infect Immun. 89:

Validation for the PE anti-mouse CD279 (PD-1) antibody can be found at the manufacturer website (https://www.biolegend.com/fr-lu/products/pe-anti-mouse-cd279-pd-1-antibody-6170) and cited in the following works:
Good-Jacobson KL, et al. 2010. Nat. Immunol. 11:535;
Tobias J, et al. 2020. Front Immunol. 11:895

Validation for the PerCP/cy5.5 anti-mouse CD366 (Tim-3) antibody can be found at the manufacturer website (https://www.biolegend.com/en-ie/products/percp-cyanine5-5-anti-mouse-cd366-tim-3-antibody-13043?GroupID=BLG10787) and cited in the following works:
Nakae S, et al. 2007. Blood 110(7):2565-8;
Oikawa T, et al. 2006. J. Immunol. 177(7):4281-7.

Validation for the PE-conjugated antihuman HLA-A, HLA-B, and HLA-C antibodies can be found at the manufacturer website (https://www.biolegend.com/fr-ch/products/pe-anti-human-hla-a-b-c-antibody-1872) and cited in the following works:
McLoughlin RM,et al.2008. J. Immunol. 181:1323;
Feola S, et al. 2021. ACS Nano. 15:15992.

Validation for the APC anti-mouse H2-Kd antibody can be found at the manufacturer website (https://www.biolegend.com/fr-ch/products/apc-anti-mouse-h-2kd-antibody-6845) and cited in the following works:
Ma XT, et al. 2006. Cancer Res. 66:1169;
Norian LA and Allen PM. 2004. J. Immunol. 173:835.

Eukaryotic cell lines

Policy information about cell lines and Sex and Gender in Research

Cell line source(s)	The murine cell line AB12 was kindly donated by Dr Lukasz Kuryk. Human cell lines JL-1 (JL1), NCI-H28 (H28), NCI-H2452 (H2452), and MSTO-211H (211H) were kindly donated by Vilja Pletiäinen and Teijo Pellinen from FIMM (the Finnish institute of molecular medicine). Patients' samples (MESO001, MESO002) were Kindly provided by Dr. Ilkka Ilonen. Human lung adenocarcinoma cell line A549 was purchased from ATCC.
Authentication	None of the cell lines used in this study were authenticated. However, immunopeptidomics analysis showed that the observed peptide HLA-binding motif for each cell line haplotype composition matched with the expectations.
Mycoplasma contamination	All cell lines were tested for mycoplasma contamination using MycoAlert™ Mycoplasma Detection Kit (Lonza) following manufacturer instructions. Murine cells were additionally tested prior in vivo tumor engraftment. All cell lines tested negative for mycoplasma contamination.
Commonly misidentified lines (See ICLAC register)	None of the cell lines used in this study were found in the registry of commonly misidentified cell lines (v11)

Animals and other research organisms

Policy information about studies involving animals; ARRIVE guidelines recommended for reporting animal research, and Sex and Gender in Research

Laboratory animals	All mice used for the this study were purchased from ENVIGO. All mice were female BALB/c aged 4-6 weeks at the moment of the delivery. Mice were housed in individually ventilated cages (IVC) for a maximum of 5 mice per cage with food and water provided ad libitum and 12h of light/dark cycle.
Wild animals	The study did not involve the use of wild animals
Reporting on sex	Findings do not apply to a only one sex.
Field-collected samples	The study did not involve samples collected from the field.
Ethics oversight	All in vivo experiments were reviewed and approved by the Experimental Animal Committee of the University of Helsinki and the Provincial Government of Southern Finland (license numbers ESAVI/11895/2019 and ESAVI/12722/2022)

Note that full information on the approval of the study protocol must also be provided in the manuscript.

Flow Cytometry

Plots

Confirm that:

- [ ] The axis labels state the marker and fluorochrome used (e.g. CD4-FITC).
- [x] The axis scales are clearly visible. Include numbers along axes only for bottom left plot of group (a 'group' is an analysis of identical markers).
- [ ] All plots are contour plots with outliers or pseudocolor plots.
- [x] A numerical value for number of cells or percentage (with statistics) is provided.

Methodology

Sample preparation	In the case of adherent cell culture, cells were detached either by scraping or by incubating them with PBS + 10mM EDTA. Cells were stained using the following procedure: detached cells were centrifuged at 600xg for 5 minutes and washed twice with PBS. Cells were then blocked using either TruStain Fc block anti-mouse (BioLegend) or anti-human CD16/32 (BioLegend, B247182) according to the respective manufacturer instructions followed by staining with fluorochrome-labeled antibodies incubated on ice for 30 minutes protected from light. Stained cells were then washed twice with PBS before sample acquisition
Instrument	BD Accuri 6C Plus Flow Cytometer
Software	Flow cytometry data was analysed with Flowjo (BD).
Cell population abundance	No cell sorting was used in this study
Gating strategy	Gating strategy for Suplementary Figure 1 was as following: Live cells based on FSC/SSC, singlets identification using FSC-A vs FSC-H; pan-HLA:PE positive population compared to unstained control.<br><br>Gating strategy for Supplementary Figure 4 was the following: Live cells based on FSC/SSC, singlets identification using FSC-A vs FSC-H; H2-Kd:APC positive population compared to unstained control.<br><br>Figure 6 and Supplementary Figure 6 were composed of 2 different flow cytometry panels. First panel was composed of the following antibodies: CD3, CD8, PD1, CD107a. Gating strategy was as following: Live cells based on FSC/SSC; singlets identification using FSC-A vs FSC-H; CD3+ from SSC vs CD3; CD8+ from CD3 vs CD8; CD107a from CD8 vs CD107a; PD1 from CD8 vs PD1; CD107a form CD8+PD1+.<br>Second panel was composed of the following antibodies: CD3, CD8, PD1, CD366 (Tim-3). Gating strategy for the second panel was as following: Live cells based on FSC/SSC; singlets identification using FSC-A vs FSC-H; CD3+ from SSC vs CD3; CD8+ from CD3 vs CD8; TIM-3 + from CD8 vs TIM-3; PD1 from CD8 vs PD1; TIM3 form CD8+PD1+.

- [ ] Tick this box to confirm that a figure exemplifying the gating strategy is provided in the Supplementary Information.
